# Supplementary material for: Eudebeiolide B Inhibits Osteoclastogenesis and Prevents Ovariectomy-Induced Bone Loss by Regulating RANKL-Induced NF-κB, c-Fos and Calcium Signaling
Source: Pharmaceuticals (Basel). 2020 Dec 16;13(12):468. doi: 10.3390/ph13120468 (PMC7765597; doi:10.3390/ph13120468)
Supplement: Supplementary file 1 [file pharmaceuticals-13-00468-s001.pdf]

pharmaceuticals

Supplementary Material

Eudebeiolide B inhibits osteoclastogenesis and prevents ovariectomy-induced bone loss by regulating RANKL-induced calcium signaling

Mi-Hwa Kim [1,†], Hyung-Jin Lim [2,†], Seon Gyeong Bak [2], Eun-Jae Park [2], Hyun-Jae Jang [3], Seung Woong Lee [2], Soyoung Lee [2], Kang Min Lee [4], Sun Hee Cheong [5], Seung-Jae Lee [2,*] and Mun-Chual Rho [2,*]

[1] Biological Resources Research Group, Gyeongnam Department of Environment Toxicology and Chemistry, Korea Institute of Toxicology (KIT), Jinju 52834, Korea
[2] Immunoregulatory Material Research Center, Korea Research Institute of Bioscience and Biotechnology (KRIBB), Jeongeup 56212, Korea
[3] Natural Medicine Research Center, Korea Research Institute of Bioscience and Biotechnology (KRIBB), Cheongju 28116, Korea
[4] Department of Molecular Biology, Chonbuk National University, Jeonju-si, Jeonbuk 54896, Korea
[5] Department of Marine Bio Food Science, Chonnam National University, Yeosu 59626, Korea
* Correspondence and requests for materials should be addressed to S.-J.L (email: seung99@kribb.re.kr) or M.-C.R (email: rho-m@kribb.re.kr)

	Contents	Pages
**Figure S1.**	Anti-osteoclastogenesis activities of sesquiterpenoids isolated from *Salvia plebeia* EtOH extract	S3
**Figure S2.**	HPLC chromatogram of eudebeiolide B and *Salvia plebeia* EtOH extract	S4
**Figure S3.**	Cytotoxicity of eudebeiolide B on MC3T3-E1 cells	S5
**Table S1.**	Total contet of compounds in ethanol extract of *Salvia plebeia* R. Br.	S6

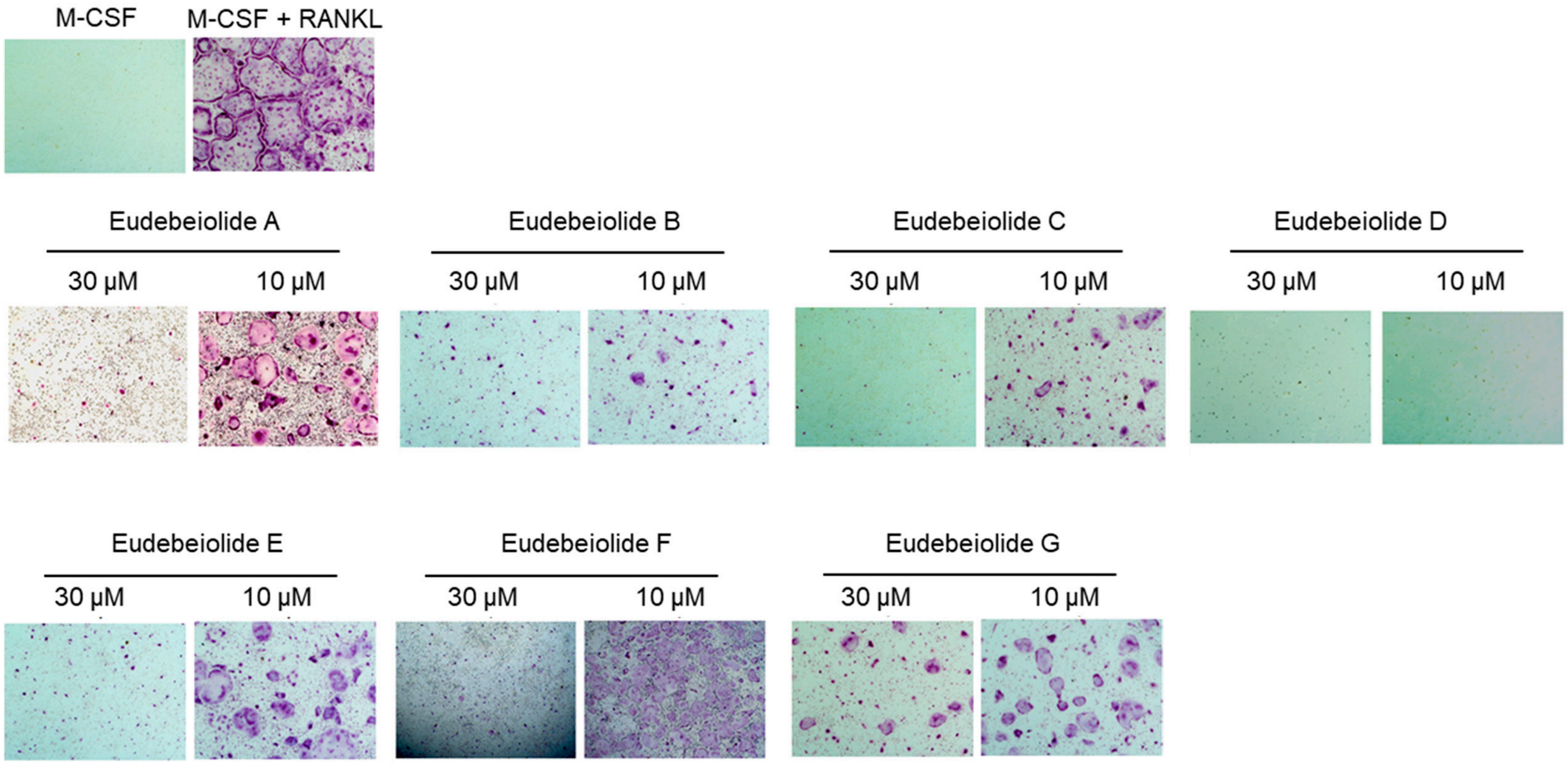

Figure S1. Anti-osteoclastogenesis activities of sesquiterpenoids isolated from *Salvia plebeia* EtOH extract.

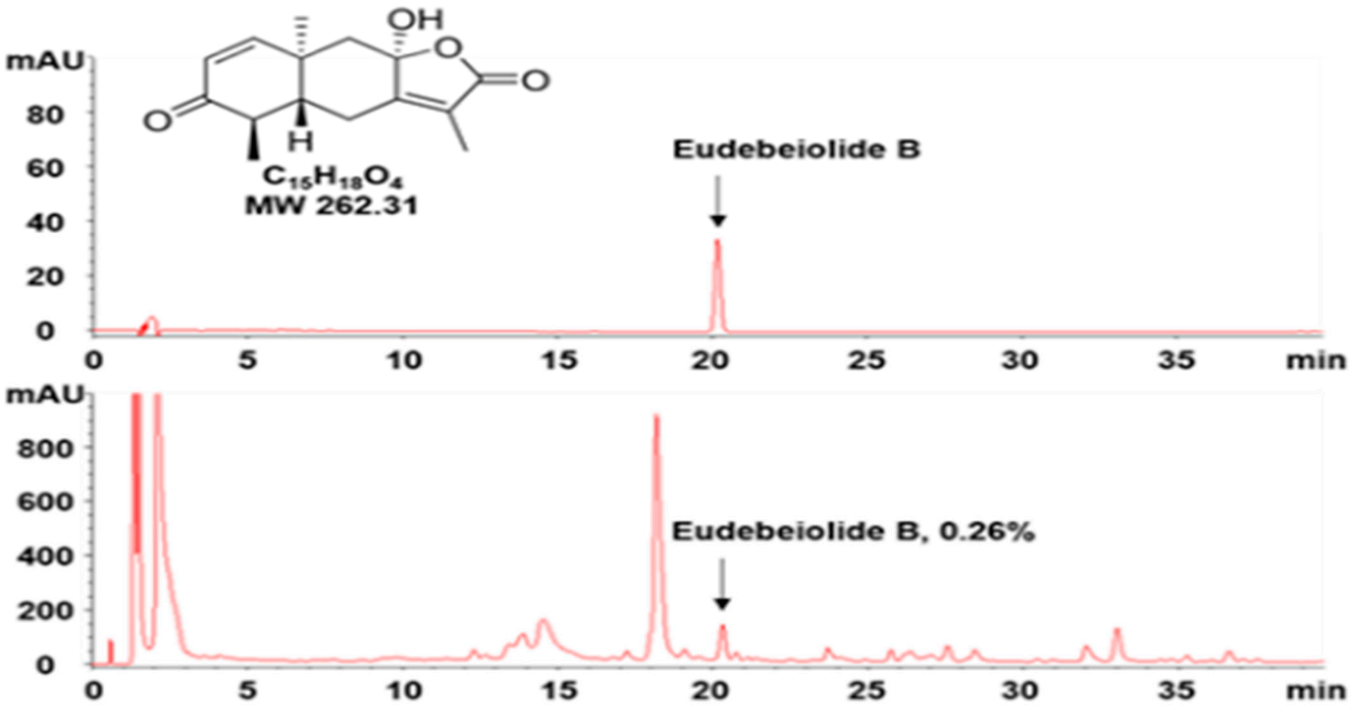

Figure S2. HPLC chromatogram of eudebeiolide B (upper panel) and *Salvia plebeia* EtOH extract (lower panel).

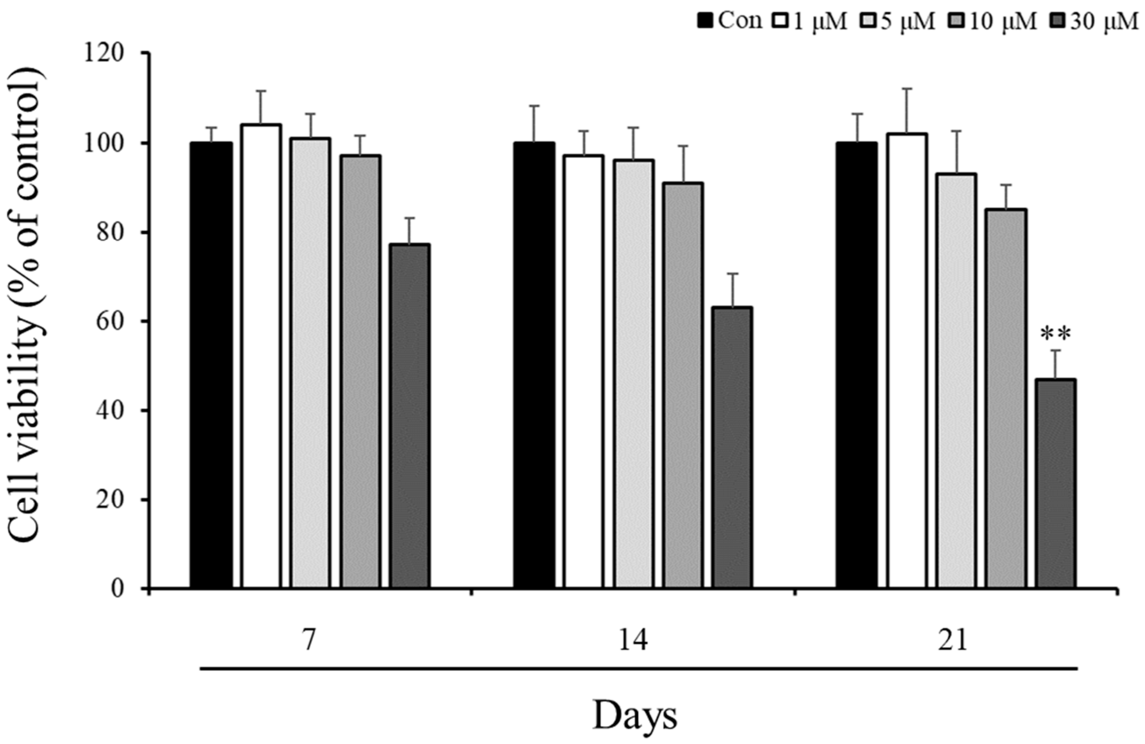

Figure S3. Cytotoxicity of eudebeiolide B on MC3T3-E1 cells. MC3T3-E1 cells were seeded in 96 well plate and treated with indicated concentration of eudebeiolide B. After incubation mtt assay was performed to obtain cell viability. Values are expressed as the means ± S.D of three individual experiments. **p* < 0.05 and ***p* < 0.01 versus control group obtained through one way ANOVA followed Dunnett's test.

Table S1. Total contet of compounds in ethanol extract of *Salvia plebeia* R. Br. (2.6 kg).

**Compound Name**	**Total Content (mg)**
Eudebeiolide A	1.9
Eudebeiolide B	24.5
Eudebeiolide C	8.4
Eudebeiolide D	12.3
Eudebeiolide E	11.4
Eudebeiolide F	2.2
Eudebeiolide G	10.9
